# Percutaneous Needle Decompression: A Novel Approach Toward Treating Compartment Syndrome of the Leg

**DOI:** 10.7759/cureus.54926

**Published:** 2024-02-26

**Authors:** Syed Abdullah Haider, Sadia Iram, Pervaiz Iqbal

**Affiliations:** 1 Medicine, University College of Medicine and Dentistry, Lahore, PAK; 2 Medicine, Lahore Medical and Dental College, Lahore, PAK; 3 Orthopedic Surgery, University of Lahore Teaching Hospital (UOLTH), Lahore, PAK

**Keywords:** spinal anesthesia, alternative treatment, fasciotomy, open fractures, acute compartment syndrome of leg, percutaneous needle decompression

## Abstract

Percutaneous needle decompression (PND) can be a successful alternative to open fasciotomies for acute compartment syndrome (ACS). We present the case of a 45-year-old male patient who survived a road traffic accident and developed ACS following his open fracture of the tibia and fibula. He was treated by performing PND on all compartments of the affected leg using a 24 gauge needle thus avoiding the complications of a double incision fasciotomy.

## Introduction

Acute compartment syndrome (ACS) is a medical emergency. It may occur in the hand, forearm, upper arm, abdomen, buttock, or the entire lower extremity. It can develop in any or all compartments of the calf or forearm as a result of trauma, burn, compression, crush injury, excessive exercise, soft tissue edema, hemorrhage, and other pathological abnormalities that lead to elevation of pressure within the myofascial compartment. It is an extremely painful condition that is not relieved even with strong analgesics. Recent epidemiological studies show that 69-75% of compartment syndrome cases are associated with fractures and 36% follow tibial diaphyseal fractures. McQueen et al. studied 850 patients and concluded that continuous intra-compartmental pressure monitoring should be considered following tibial diaphyseal fracture because these patients are at risk for ACS [1]. Feliciano et al. reported the frequency of compartment syndrome is much higher in patients who have an associated vascular injury [2]. It is a fallacy that ACS doesn’t happen in open fractures, evidence suggests that 15% of patients with open fractures develop ACS later. However, the occurrence rate of ACS is higher in closed injuries as it causes hematoma and compresses the major vessels further aggravating the ischemia. If allowed to progress, untreated compartment syndrome can lead to gangrene, chronic muscle contractures (Volkmann contracture), paresthesia, paralysis, infection, and non-union of fractures. Approximately 1-10% of patients of ACS develop a Volkmann contracture [3]. Muscle necrosis releases myoglobin which is excreted in urine, damaging the kidney and leading to renal failure. Calcific myonecrosis of lower extremity muscles has been identified as an uncommon late complication of posttraumatic compartment syndrome. Another study suggests that there was a significant delay in tibial union in the people who were not monitored for ACS [4].

In the lower leg, peroneal nerve palsy may develop. The compartment syndrome may follow operations for orthopedic fixation. These cases may result from postoperative hematoma, muscle edema, or tight closure of the deep fascia. Excessive traction (raises pressure in the deep posterior compartment by approximately 6% per kg of weight applied) and high elevation of the leg should be avoided in patients at risk for ACS.

Early diagnosis is very crucial and high-risk patients should be monitored closely especially unconscious patients. The diagnosis may be clinical or by measuring the intra-compartmental pressure. Excruciating pain on passive extension of toes or fingers is a common and important early sign of ACS in the calf and forearm, respectively. The patient may have a sensory deficit in the distribution of nerves in that compartment. Direct intra-compartmental pressure measurements using the Whitesides technique or slit catheter technique at the fracture site provide an accurate assessment of compartment conditions. If the compartmental pressure is more than 30 mmHg for more than two hours, emergent compartment release is required [5]. Non-invasive methods for diagnosis of ACS are being examined such as near-infrared spectroscopy which measures the amount of oxygenated hemoglobin in muscle tissue transcutaneously [6].

Delay in diagnosis has been cited as the only reason for failure in management of ACS and it places the surgeon at high risk for litigation. A delay in fasciotomy of more than six hours can cause significant damage due to muscle and nerve ischemia. If fasciotomy is delayed beyond 12 hours, only 8% of patients showed a return to normal function. Tendon transfers and stabilization may be indicated as late treatment for CS. In severe cases, amputation may be necessary. If the duration of compartment syndrome exceeds over 72 hours, fasciotomies should not be performed as it will expose dead necrosed muscle and increase the risk of infection.

Percutaneous needle decompression (PND) is a minimally invasive medical procedure used to relieve pressure or tension within a body cavity or a closed space by making needle punctures through the skin. This technique is commonly employed in various medical specialties, including neurosurgery, orthopedics, and interventional radiology. In orthopedics, PND has been previously used to treat conditions such as bursitis and ganglion cysts. Its use in the treatment of compartment leg syndrome is rather new and not very widely known. The objective of this case report is to present a case in which a patient with compartment leg syndrome was successfully treated by PND. It's important to note that while PND can be an effective treatment option for this condition, its suitability depends on various factors such as the patient's medical history, the severity of the condition, and the expertise of the healthcare provider performing the procedure.

## Case presentation

The patient was a 45-year-old male. He had a road traffic accident on November 24, 2022. The patient suffered a comminuted grade 3 open fracture of the tibia and fibula. Fracture reduction and other initial management were done as emergency treatment in the nearby clinic, IV fluids, and anti-tetanus serum were administered along with antibiotics.

The patient was admitted through emergency and was referred to the outpatient department at the ortho department of the University of Lahore Teaching Hospital, Lahore, Pakistan, on November 25, 2022, with the complaint of pain, tenderness, and restricted range of motion of the left leg. He was diagnosed with compartment leg syndrome clinically. The diagnosis was confirmed using the Whiteside technique upon which the compartment pressure was found to be higher than 30 mmHg. Doppler was performed to rule out any vascular injury. All the preliminary necessary investigations were done including CBC, international normalized ratio, prothrombin time, activated partial thromboplastin time, renal function tests, anti-hepatitis C virus, hepatitis B surface antigen (HBsAg), and chest X-ray, and no significant findings were observed. There was no other major injury on the body or any associated systemic complaint.

**Figure 1:**
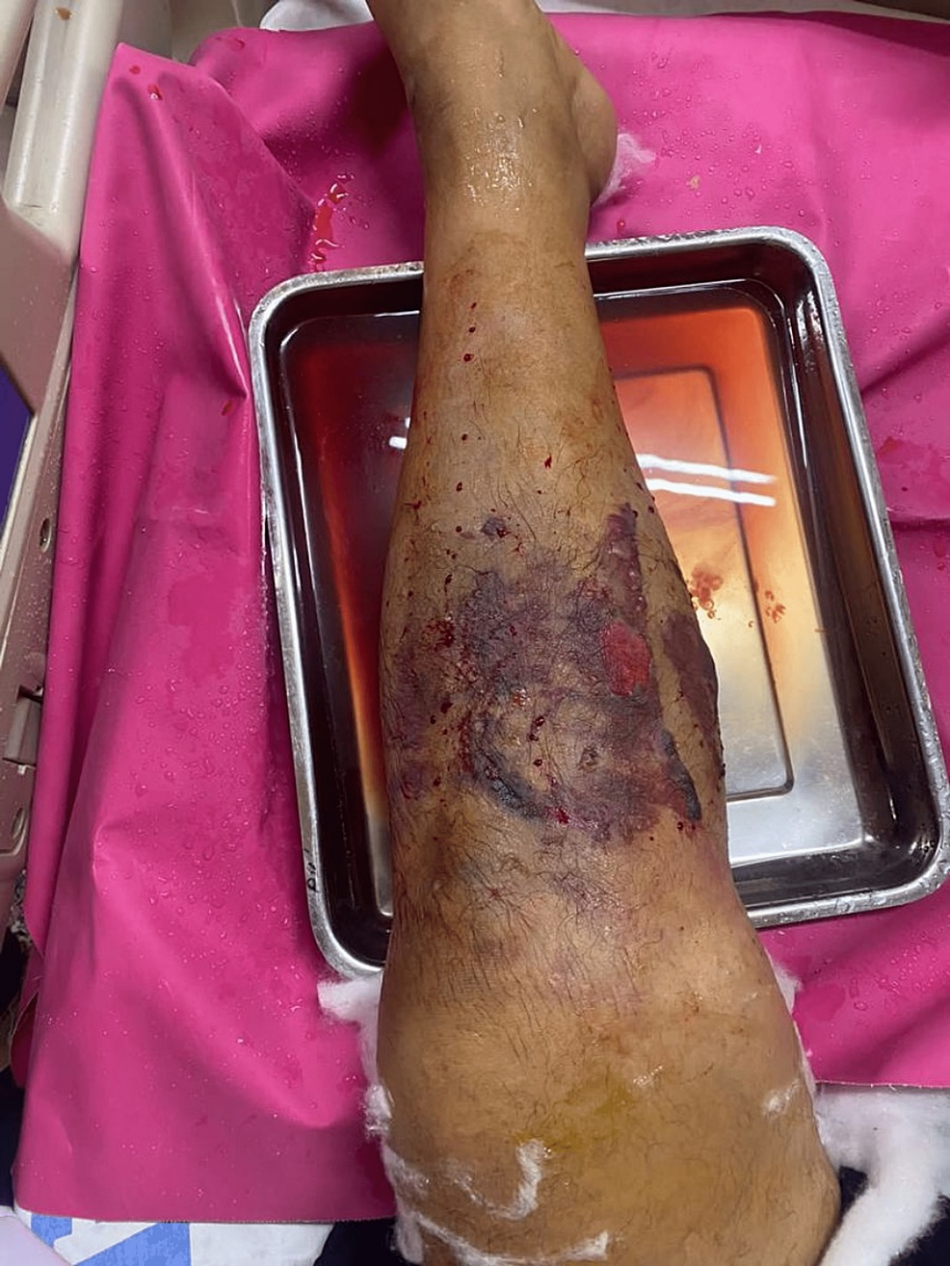
Percutaneous needle decompression being performed on the left leg of the patient. The needle punctures can be clearly appreciated.

**Figure 2:**
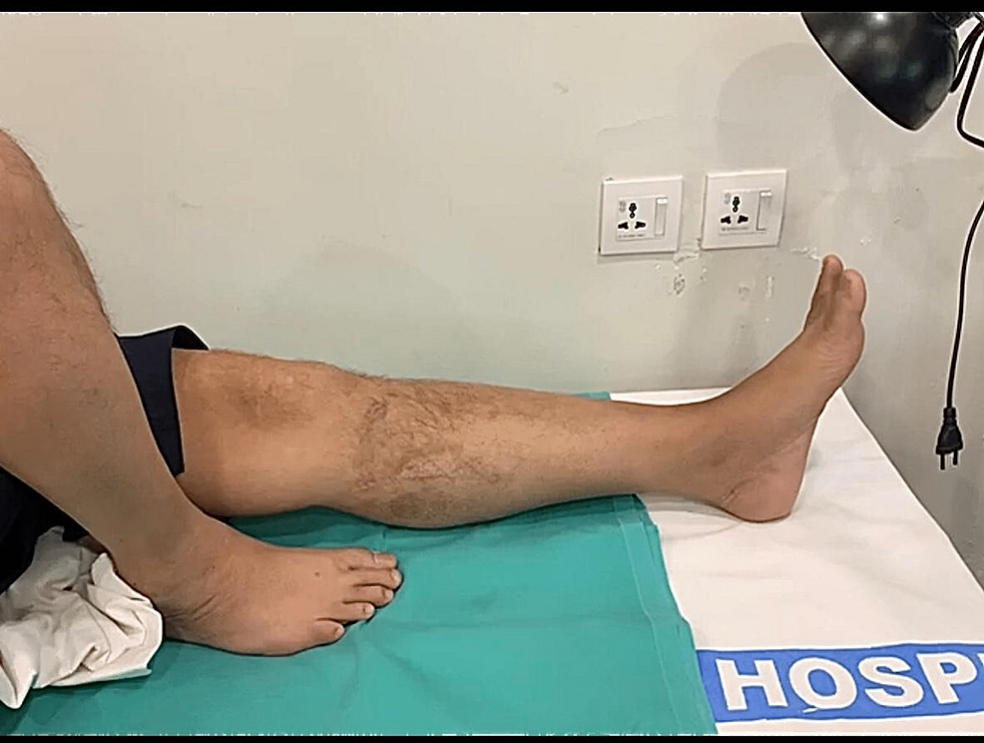
Follow-up examination of the patient after two months. No significantly visible scarring from the procedure was noticed.

The patient underwent PND following informed written consent. The treatment was approved by the ethical committee of the hospital. The procedure was performed on November 26, 2022, under spinal anesthesia soon after confirmation of the diagnosis. The technique involved administering approximately 200 punctures around the circumference of the leg in a vertical manner, about 20 punctures in each arbitrary line with the help of a size 24 gauge needle under an aseptic technique avoiding vital structures (Figure 1). The blood came out gradually by oozing from needle punctures, vertical padding was done, then an aseptic dressing was applied and the leg was elevated on a pillow during the postoperative period. Triple padding was done for the initial four days post-procedure, with subsequent adjustments in frequency as deemed necessary. The patient was kept under observation for two days in the ward. The compartments were significantly decompressed within 12 hours to 24 hours and completely decompressed in 48 hours. The intra-compartmental pressure dropped to normal and was monitored using the Whiteside technique after 48-72 hours of the procedure. Conventional post-op antibiotics and non-steroidal anti-inflammatory drugs were prescribed. He was kept on follow-up till three months after discharge. Upon follow up he was evaluated for any recurrence of symptoms, healing progress, signs of infection, and any vascular or neuronal complication. Minimal scaring was observed on follow-up visits (Figure 2).

## Discussion

Although fasciotomy is the standard procedure used around the world for the treatment of acute compartment leg syndrome, PND has been brought forward as a new technique with similar yet better results. A case series has been published before regarding 30 patients with ACS who were treated using this procedure successfully [7].

Fasciotomy is a surgical procedure in which the skin incision is along the full length of the affected compartment. Fasciotomy incisions are not closed primarily because this may result in persistent elevation of intracranial pressure; therefore, a second surgery is usually required. If delayed primary closure can’t be achieved, the wound may be closed by dermato-traction techniques or split skin grafting. Dermato-traction techniques may cause skin edge necrosis [8]. A further disadvantage is the prolonged time required for closure, which is up to nine days. Split skin grafting, although it offers immediate skin cover, has a high rate of long-term morbidity [9]. On the contrary, in PND, the needle pricks are hardly visible after about 72 hours.

In the lower leg, the results of fasciotomies for the posterior compartment are not as good as those for the anterior compartment, as it is difficult to completely decompress the deep posterior compartment because of the morbidity associated with this procedure [10]. Although PND is equally effective in releasing the posterior compartment of the leg, infection is a serious complication of compartment syndrome. In a retrospective review by Finkelstein et al., five out of nine patients who had late surgical decompression developed infections and septicemia that further led to amputation and one patient died of multiorgan failure [11]. Hence, another edge of PND is that the critical time of the patient is saved as this procedure can be performed in the emergency room under aseptic techniques and doesn’t require much expertise; hence, it can be performed by a trainee as well. PND normalizes the compartment pressure within 24 to 48 hours of the procedure but in case of failure to achieve the desired results the procedure can be reperformed.

The only known limitation of PND in the treatment of compartment syndrome is that it cannot be performed in cases where ACS is caused by a vascular injury. To rule this out doppler should be performed before the initiation of treatment. In case of ACS caused by vascular injury, the surgeon should opt for fasciotomy. It is not a widely used and well-known procedure to treat compartment leg syndrome, so further research needs to be done on a larger scale, although no major complications have been observed so far.

## Conclusions

This case report reinforces that PND may be proven as a better, less invasive treatment for compartment leg syndrome, as it seems superior to fasciotomy in a lot of comparative points. It bypasses incisions and complications of reconstruction of muscles such as nerves or vessel gangrene. Thus, it provides a better and immediate recovery, even though the procedure itself is not time-consuming at all. Such new, better-advanced treatments with fewer complications and faster recovery should be introduced as standard practice, and doctors must be efficiently trained and equipped for such procedures. We recommend that PND may be considered in patients with ACS of the leg as an alternative to double-incision fasciotomy of the leg.
